# Mistreatment of Women in Childbirth: Time for Action on This Important Dimension of Violence against Women

**DOI:** 10.1371/journal.pmed.1001849

**Published:** 2015-06-30

**Authors:** Rachel Jewkes, Loveday Penn-Kekana

**Affiliations:** 1 Gender & Health Research Unit, Medical Research Council, Pretoria, South Africa; 2 School of Public Health, University of the Witwatersrand, Johannesburg, South Africa; 3 Maternal Health Group, Department of Infectious Diseases and Epidemiology, London School of Hygiene & Tropical Medicine, London, United Kingdom

The mistreatment of women in childbirth has been documented by researchers for over three decades in all global regions. The scale of the problem is indicated by a systematic review conducted by Meghan Bohren and colleagues [1], which provides a foundation from which a typology of violence can be developed and used as a basis for developing measurement instruments and tools. This is a valuable complement to other work that is currently underway in this area [2]. A multicountry study on the mistreatment of women during childbirth could be extremely valuable in generating comparable information on prevalence, risk groups and facilities, and the health consequences (physical and mental, including future health-seeking practices and expectations). It would provide the foundation needed for developing health policy, monitoring its impact, and advocating for proper resources.

## Mistreatment of Women in Childbirth as a Subset of Violence against Women

From Bohren and colleagues’ systematic review, it is very easy to draw parallels between the mistreatment of women in childbirth and violence against women more broadly, and these parallels may lead us to conclude that the former should indeed be viewed as a further subset of the latter. The essential feature of violence against women is that it stems from structural gender inequality, i.e., women’s subordinate position in society as compared to men. This systematically devalues the lives of women and girls and thus enables the inappropriately low allocation of resources to maternity care that is found in many countries [3]. It also disempowers women and enables the use of violence against them.

The power relations between some health professionals and women in maternity settings are ones of hegemonic dominance, which strongly parallels the societal position of dominance of men [4,5]. Negative behaviour largely stems from social norms within these environments, which influence both practice and expectations of power and are largely taken for granted [5]. This can lead to the expectation that staff will be in control of women patients and entitled to use a range of strategies, including physical violence, to achieve this control and punish perceived disobedience [6]. Female patients largely have little choice but to acquiesce to the power of the professionals in this setting, as they feel very vulnerable, especially without birth companions who could be advocates for them. The lack of repercussions for unacceptable health worker behaviour can fuel a sense of entitlement [6].

Many of the health professionals who act most harshly towards women perceive themselves to be disempowered, as they work in less glamorous parts of the health system (such as rural clinics in low-resource settings), are often low paid, and may experience abuse at home as women [6]. Their abusive behaviour may in effect be compensatory for the lack of perception of power in other areas of life, as has been reported for some violent men [6,7]. However, this does not make their abusive behaviour any less of a problem for affected women. It may not be a coincidence that the types of mistreatment women experience at the hands of health professionals overlap greatly with the types of mistreatment from intimate partners or others with positions of power in the home, such as mothers-in-law. There are also likely to be many lessons learned from research on violence against women in developing policy and interventions to prevent mistreatment of women in childbirth.

## Power of Measurement

International policy development to end violence against women has hugely benefitted from research. Efforts to end the mistreatment of women in childbirth could be advanced by a similar body of work, but how it is done is important. One of the lessons from research on violence against women is that poorly drafted survey questions administered by poorly trained and supported teams can underestimate the prevalence of violence [8]. Mixed methods may be needed in research, with women’s self-reports of mistreatment complemented by findings from direct observation [9]. Those administering questionnaires need to be knowledgeable about the subject of the research and well trained in empathetic interviewing. They should also be equipped to support women if they become distressed after talking about their experiences. Confidentiality is also critical, as women may fear repercussions in small communities for disclosure of abuse. Ethical and safety guidelines should be developed for this field, as they have for others [10].

Bohren and colleagues’ systematic review is organised into a typology of six forms of violence, with some overlap between the categories and presentation of a large range of diverse acts [1]. A more focused definition may be beneficial. There are two underlying dimensions to the problem: intentional use of violence—physical abuse, verbal abuse (including that stemming from stigma), and negligent withholding of care—and structural disrespect, which is reflected in deviations from accepted standards for infrastructure, staffing, equipment availability, and supplies needed to deliver care, as well as in unnecessary interventions, demands for illegal payments, and the detainment of people in facilities until they have paid their bills.

Keeping a focus on both of these dimensions is important. The field of advancing respectful maternity care has already encountered considerable hostility to the idea that women are “abused” by staff, with some objectors arguing that health workers are already demoralised and alienated. Given the evidence, however, it is important that intentional mistreatment is kept firmly centre stage through the drafting of the definition, as it would be a pity if a door was opened to enable a roundup of the “usual suspects” for interventions to improving maternity care instead of focusing on the highly uncomfortable area of deliberate mistreatment [11].

## Development of Policy and Practice to Prevent Mistreatment of Women in Childbirth

Prevention needs to be built on a solid understanding of the root causes, some of which have been elucidated through qualitative research, but more work is needed [6]. Inevitably, interventions are required at multiple levels [12]. Political will to address the problem will need to be built among the professions as well as governments, and resources will need to be allocated. There is a need for institutional policies, supported by resource allocation, training and supervision of staff, and enforcement. Work with men on building gender equity suggests that staff who do not mistreat women may be engaged as allies in change processes if the right approach and methods are used [13]. It is possible to engage health workers in processes of critical reflection on building understanding and empathy [14] and show through experiential learning the rewards in terms of job satisfaction that stem from more positive relationships. Engagement needs to be approached without “blaming health workers” as a group, as evidence points to the widespread impact of social norms of care on individual-level practices; thus, it is more constructive to work on overall improvement in practices whilst holding individuals accountable for more severely abusive actions [2]. In this respect, existing interventions such as “Health Workers for Change” may be very valuable [15]. Women can also be supported in their roles in childbirth by being provided with better antenatal education and information, something which is sorely missing in many settings, and help in understanding their rights. Birth companions have been shown to improve maternal outcomes and might have a substantial impact on reducing mistreatment of women in childbirth.

There is no doubt that it is within the power of societies and their health sectors to end the mistreatment of women in childbirth. There is a need to much better understand the nature of the problem and its consequences and to use research to develop the political will to eradicate it.
